# Supplementary material for: Getting More Out of Biomedical Documents with GATE's Full Lifecycle Open Source Text Analytics
Source: PLoS Comput Biol. 2013 Feb 7;9(2):e1002854. doi: 10.1371/journal.pcbi.1002854 (PMC3567135; doi:10.1371/journal.pcbi.1002854)
Supplement: Dataset S1 — GATE software. Dataset S1 bundled with this paper contains a distribution of GATE (or see http://gate.ac.uk/download/). (TGZ) [file pcbi.1002854.s001.tgz › plos-gate/src/gate/resources/gate.ac.uk/tests/in-outlook-09-aug-2001.html]

Independent News


<a href="http://west.adlink.de/adserver/accipiter/adclick.exe/UKCATEGORY=ECOMMERCE/SITE=UK\_IDUK\_UK/AREA=NEWS.BUSINESS.OUTLOOK/AAMSZ=468X60/ADTYPE=GIF/ACC\_RANDOM=576873786" target="\_blank">
<img src="http://west.adlink.de/adserver/accipiter/adserver.exe/UKCATEGORY=ECOMMERCE/SITE=UK\_IDUK\_UK/AREA=NEWS.BUSINESS.OUTLOOK/AAMSZ=468X60/ADTYPE=GIF/ACC\_RANDOM=576873786" border="0" width="468" height="60" alt="Click here!"></a>


News | 
Money | 
Travel | 
Argument | 
Advancement | 
Enjoyment

Independent > 
News
 > Business
 > Outlook


# The windfall that would reap a bitter harvest

## Fuel tax; Pensions poser; Sir Stanley's fury

#### 09 August 2001

**Internal links**

Today's young must not become the poverty-struck pensioners of tomorrow

New Labour think-tank warns Blair that pensions strategy in danger of 'unravelling'

It does not suit the tabloid agenda to say so, but there is already a windfall tax on petrol and it is called duty and VAT. For every £1 the motorist spends filling up at the pumps, 76p goes straight into the pocket of Gordon Brown. It is for this reason, and no other, that the UK has the highest fuel prices in Europe. The motoring lobby, however, prefers to believe it is all the fault of profiteering oil companies and is baying for Shell and BP to be penalised for the "obscene" profits they are making ­ £1.3m an hour since you ask.

What would happen to the windfall tax levied on these North Sea profits? The subtext is that the Chancellor should recycle the money so we can enjoy lower fuel prices. But that would make a mockery of the Government's environmental credentials.

Taxing away the meagre profits oil companies make at the pumps would have almost no impact on the cost of a gallon. But imposing a super-tax on the profits they make from oil exploration would have all sorts of harmful repercussions.

Oil companies are internationally mobile and if the tax regime in the UK became harsher, they would vote with their feet and take their investment elsewhere. Mondeo Man would not notice any difference when he filled up. But the hundreds of firms and thousands of workers who rely on the North Sea for a living most certainly would.

BP makes only 17 per cent of its profits in the UK, and virtually nothing from petrol retailing, which has been turned into even more of a cut-throat business since the supermarkets decided to turn it into a loss leader. Taxing those profits more heavily would be a supremely irrational act. It is profits which keep workers in jobs and pay the dividends which fund everyone's private pension. And what would the Government then do when the oil price falls and the likes of BP find themselves making barely enough money to cover their cost of capital? Pay the money back?

The changes to the North Sea taxation regime introduced over the last two decades have been hugely beneficial. Investment has flowed in, production has risen and although the direct tax take has fallen, the Exchequer benefits much more from the corporation tax paid by businesses which would otherwise not exist.

Labour came into office in 1997 keen to tighten the screw on the oil companies. But it backed off when the oil price fell and it realised that higher taxes would be an electoral liability in Scotland. Three months into its second term, renewed talk of a windfall tax is a silly season story which the markets rightly ignored. If Labour did the unimaginable and imposed one, folly would be a more apt description.

**Pensions poser**

If a windfall tax is a daft idea, then the stakeholder pension may not prove to be very much better. Yesterday Tony Blair's favourite think-tank, the Institute for Public Policy Research, took the Government's entire strategy towards pensions and long-term care for the elderly to task, suggesting it might be "unravelling".

A spokeswoman for the Department for Work and Pensions bit her lip, welcomed this "valuable contribution to the debate" and went away with a wet towel around her head to analyse the IPPR's recommendations.

Its central conclusion is one that many observers came to even before the stakeholder pension was launched ­ that it is probably going to miss its target group, who currently have only the basic state pension to look forward to in retirement.

These are people who cannot afford to pay into a private pension in the first place. They are unlikely to make the extra effort simply because charges are capped at 1 per cent. But the cap on commissions is one more reason why stakeholders are so attractive to well-heeled grandparents looking a tax-efficient way to pass capital on to their grandchildren.

The IPPR does not stop at stakeholders, however. It goes on to criticise the Government's enormously complicated approach to tackling pensioner poverty through a battery of pension credits, minimum income guarantees and second state pensions.

The pot is only so big and a generous increase in universal state provision after the public relations disaster of the 75p pension increase, can only come at the expense of providing incentives to save.

Pension provision is only one side of the coin. The other is long-term health care for the elderly. The present system penalises those who save for retirement by making them pay for their care. This is a subject successive governments have shied away from tackling. The IPPR report at least comes up with some options ­ such as allowing the financial services industry to use pensions as a vehicle for providing long-term care.

There is a trade off. Enabling the elderly to live off a bigger income in retirement may be one way of extending their independence and delaying the day when they become reliant on formal long-term care and a drain on the public purse. That, however, would require joined-up thinking from a joined-up government.

**Sir Stanley's fury**

No one could ever accuse Sir Stanley Kalms of being backward in coming forward and, sure enough, the ebullient Dixons chairman is on the offensive again. As a rabid Tory supporter and ferocious anti-euro campaigner his targets are familiar. In the firing line once more is increasing red tape at home and Brussels bureaucracy abroad.

His latest attack is contained in Dixons' annual report and given the strength of his views perhaps the only surprise is that he managed to restrict himself to a single paragraph on the subject. Even so he managed to pack in enough punches, saying Dixons was battling against "the most severe and costly regulation the market economy has experienced both from Brussels and our own Government". The demands are often "absurd" and "unworkable", he says, and serve only "to add costs which are eventually borne by the consumer". Phew. Just as well the management got a 10 per cent pay rise and all those lovely share options to compensate for the extra demands on them.

Sir Stanley doesn't mention specific examples but Dixons was only too happy to provide some yesterday. Its chief target is a draft EU directive on waste from electrical equipment that will be put to the Council of Ministers next month. Its aim is to encourage greater recycling of old electrical products by making electrical retailers take back defunct goods in their shops. Dixons says this will mean expensive warehousing and training for its staff, not to mention clapped-out TVs being dumped on the floor at the local Dixons, which will hardly be good for trade. The claim is that the proposed directive will cost Dixons millions and affect 120,000 UK businesses from department stores and supermarkets to thousands of small specialist operators.

Sir Stanley's great fear is that this piece of red tape from a bunch of Euro-suits will eat into his profits. You can almost see him throwing a brick through the telly in his fury.

m.harrison@independent.co.uk

### Also in Outlook

No end in sight yet to the tech stock meltdown  
The windfall that would reap a bitter harvest  
The Equitable solution to a pensions nightmare  
Chill wind starts to blow towards service sector  
BA and AA fasten their seat belts for a bumpy ride  
  


---


Legal | 
Contact us | 
Using our Content | 
c 2001 Independent Digital (UK) Ltd
